# Genome-wide analysis of Atlantic salmon (*Salmo salar*) mucin genes and their role as biomarkers

**DOI:** 10.1371/journal.pone.0189103

**Published:** 2017-12-13

**Authors:** Lene Rydal Sveen, Fabian Thomas Grammes, Elisabeth Ytteborg, Harald Takle, Sven Martin Jørgensen

**Affiliations:** 1 Department of Biology, Section of Marine Developmental Biology, University of Bergen (UiB), Bergen, Norway; 2 Division of Aquaculture, Section of Fish health, Norwegian Institute of Food, Fisheries and Aquaculture Research (Nofima), Ås, Norway; 3 Centre for Integrative Genetics (CIGENE), Department of Animal and Aquacultural Sciences (IHA), Faculty of Life Sciences (BIOVIT), Norwegian University of Life Sciences (NMBU), Ås, Norway; Xiamen University, CHINA

## Abstract

The aim of this study was to identify potential mucin genes in the Atlantic salmon genome and evaluate tissue-specific distribution and transcriptional regulation in response to aquaculture-relevant stress conditions in post-smolts. Seven secreted gel-forming mucin genes were identified based on several layers of evidence; annotation, transcription, phylogeny and domain structure. Two genes were annotated as *muc2* and five genes as *muc5*. The *muc2* genes were predominantly transcribed in the intestinal region while the different genes in the *muc5* family were mainly transcribed in either skin, gill or pyloric caeca. In order to investigate transcriptional regulation of mucins during stress conditions, two controlled experiments were conducted. In the first experiment, handling stress induced mucin transcription in the gill, while transcription decreased in the skin and intestine. In the second experiment, long term intensive rearing conditions (fish biomass ~125 kg/m^3^) interrupted by additional confinement led to increased transcription of mucin genes in the skin at one, seven and fourteen days post-confinement.

## 1. Introduction

The mucus matrix that lines the epithelia of all the mucosal tissues has an important but poorly understood role in protection. The main constituent of the mucus matrix is the large gel-forming glycoproteins called mucins [1]. Most knowledge regarding mucins comes from studies in mammals. Intensive research on mammalian mucins is due to the involvement of mucins in intestinal protection [2], cancer [3] and diseases of the respiratory system [4].

In commercial aquaculture, fish are exposed to more stressful events than their wild relatives, which may make them more susceptible to diseases [5–7]. The route of infection is usually through the skin, gill or gastrointestinal regions, which are the main mucosal tissues. These tissues contain mucous producing cells which secret a protective mucus matrix that cover the epithelial surfaces. The matrix protects the surface both by its physical properties and by acting like a medium, facilitating the action of defense molecules and other biologically active substances [8]. The number of mucous producing cells has been suggested to reflect the health status of the mucosal tissues [9]. Increased number of mucous cells in the skin have been seen in response to external stressors such as reduced pH [10, 11] high nitrate and low O_2_ levels [12], aluminum exposure [13] and presence of pathogens [14]. Increased number of mucous cells are also reported in gills in relation to various stressful conditions such as presence of exotoxins [15] and changes in water quality [16]. Despite several publications related to mucous cell number and the protective role of the mucus layer, little knowledge exists about the mucin-encoding genes in fish. Recently, Perez-Sanchez et. al. (2013) demonstrated that intestinal mucin transcription responded to both diet and parasite infection, suggesting that mucins may be used as genetic markers for fish intestinal health.

In humans and higher vertebrates, more than twenty different mucin genes have so far been identified [2], while eleven different gel-forming mucins have been found in the model fish species Zebrafish (*Danio rerio*) [17]. The mucin proteins are separated into two functional classes: The secreted gel-forming mucins and the membrane bound mucins. The present study focuses on the gel-forming mucins.

The secreted gel-forming mucins are characterized by the presence of several domain structures; von Willebrand D (VWD), cysteine rich (C8) and trypsin inhibitor like cysteine rich (TIL) domains. These domains contribute to oligomerization of the mucin proteins through disulfide bond formation [18], which provides the gel-forming properties of mucus [19]. In addition, some mucins also have a C-terminal cysteine knot (Cys-Knot) domain, von Willebrand C (VWC) domain and the domain Mucin2_WxxW [17]. In humans, MUC2, MUC5AC and MUC5B all have the domain architecture (VWD-C8-TIL)-(VWD-C8-TIL)-(VWD-C8-TIL)-PTS-(VWD-C8-TIL) [17]. In addition, the secreted gel-forming mucins have long segments of highly repetitive sequences that are rich in proline, threonine and serine residues, referred to as the PTS-domain [20]. The number, length and amino acid sequence of the PTS domains varies among the mucins and these domains are poorly conserved among species [21]. The serine and threonine residues are sites for O-linked glycosylation which provides rigidity and solubility to the protein [18].

In Atlantic salmon, three isotigs have been predicted that exhibit homology to the mammalian mucins MUC2, MUC5AC and MUC5B; however due to short sequences, no definite identification was made in the published research [22]. As several authors have reported, the large size and the repetitive nature of the mucin genes makes their identification difficult [17]. In addition, several other non-mucin proteins also contain the VWD domain and the domain structure VWD-C8-TIL; confounding verification that the identified gene encodes an actual mucin [21].

In the current study, identification of putative mucin genes in the Atlantic salmon genome was based on several layers of evidence including domain architecture, phylogeny and transcription patterns from public RNAseq data. In addition, transcription patterns of the identified mucin genes were analyzed with quantitative real time PCR (qPCR) in mucosal tissues from healthy Atlantic salmon. Lastly, two experiments were conducted to investigate how the identified mucin genes respond to external stressors that are relevant in the commercial production of Atlantic salmon.

## Materials and methods

### Bioinformatics

#### Annotation and sequences

All sequences annotated as mucins were extracted from the Atlantic salmon genome (NCBI Reference Sequence Database (RefSeq) assembly accession: GCF_000233375.1), in total 144 sequences. PFAM domains for all protein coding genes were identified by querying each predicted protein sequence against the Pfam-A.hmm database, using hmmscan [23] (version 3.1b). As the Pfam-A.hmm database does not contain PTS domains, the PTS domains were identified using an in house R-script according to [24]. In brief, a sliding window algorithm was used to identify regions containing > 40% serine or threonine and ≥ 5% proline, using a window size of 100 amino acids. Sequences containing a domain structure of minimum VWD-C8-TIL were considered to be potential mucin candidates. Out of the 144 sequences 25 had this domain structure.

#### RNAseq data

A publicly available RNAseq dataset spanning 9 different tissues (Sequence Read Archive: PRJNA260929) [25], was used to evaluate transcription for all the identified potential mucin or mucin-like genes (144 genes). In total, 52 out of the 144 sequences were transcribed in one or more tissue.

#### Domains and phylogeny

The phylogenetic tree was built by aligning the conserved VWD domains. The sequences of the VWD domains, hereafter referred to as D1-D4 based on their relative position in the protein starting from the N-terminus, were used in the alignment. Public available mucin sequences from other species were downloaded from Ensemble [26], (S1 File). Protein sequences from all VWD domains were extracted and numbered in sequence from the N-terminus. The domain sequences were aligned using mafft (v7.215) [27]. The alignments were subsequently imported into R where the phylogenetic tree was built using a maximum likelihood model (*optim*.*pml* function implemented in the “phangorn” R package [28], using the WAG substitution model for AA[29]). Branch support was evaluated by bootstrap analysis based on 100 pseudoreplicates using the *bootstrap*.*pml* function [28]. An in-house R-script was used to build domain structures for the Muc2 and Muc5 families from the downloaded sequences.

### Fish experiments and tissue collection

#### Tissue-specific transcription study

**Fig 1:**
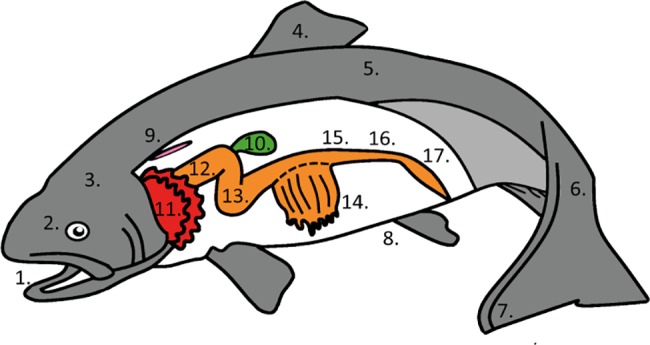
Tissue sections for mucin transcription analyses with quantitative real time PCR. 1. Eye 2. Tongue 3. Operculum 4. Dorsal fin 5. Dorsal skin 6. Caudal skin 7. Caudal fin 8. Ventral skin 9. Ovarium 10. Gall bladder 11. Gill 12. Esophagus 13. Stomach 14. Pyloric ceca 15. Anterior intestine 16. Middle intestine 17. Posterior intestine.

Post-smolts (SalmoBreed strain, Norway) were reared in 500 L square fiberglass tanks (25 kg/m^3^) under standard conditions in a flow-through system with 32 ppt seawater at Nofima’s Centre for Recirculation in Aquaculture station (Sunndalsøra, Norway) from May 2015. Temperature was 12°C and O_2_ saturation > 80%, measured daily in the outlet water. Fish were fed Skretting Spirit Supreme 75 until July, when feed was changed to Ewos Opal 200. Fish were fed continuously from automatic feeders. On the 17^th^ of August 2015 samples were collected from fish (n = 3) with an average body weight of 300 g. Samples were collected from skin (operculum, dorsal, ventral and caudal side of the body), fins (dorsal and caudal fin), eye, tongue, esophagus, stomach, intestine (anterior, middle and posterior parts), pyloric caeca, gallbladder and ovarium (Fig 1).

#### Stress experiments

Two experiments were conducted for studies on mucin transcription following stress conditions; 1) acute short-term handling stress, 2) intensive rearing with high fish density followed by transient acute confinement stress.

Experiment 1: The same post-smolts were used as described above (tissue-specific transcription study). Control samples (n = 15) were collected prior to stress treatment. Fish were individually netted and exposed to air for 30s followed by 60s recovery in water that was continuously oxygenated (Tetratec APS 300, Southampton, UK) with O_2_ levels > 95%. This procedure was repeated three times. The fish were then transferred to the original tank and samples from skin (dorsal side), gill and middle intestine were collected 3 h and 24 h post stress.

Experiment 2: This study was carried out at the Industrial Laboratory (ILAB, Bergen Norway) between October 4^th^ and December 15^th^, 2014. Smolts (mean size 80 g) were distributed randomly among in 1 m^2^ square fiberglass tanks (500 L). The fish were stocked at a density of 125 kg/m^3^ in triplicate tanks. From the 4^th^ to the 6^th^ of October, the fresh water in each tank was gradually replaced with seawater (12°C). Following transfer to full strength seawater, the fish were exposed to a simulated natural light regime (60^o^25`N). The fish were fed a commercial dry diet (EWOS, Oslo, Norway) in 10% excess between 09.00–10.00 and 15.00–16.00 throughout the study. Temperature and O_2_ levels as described above. The experimental period started on the 28^th^ of November and lasted until the 15^th^ of December. On the 1^st^ of December the fish were exposed to confinement stress by lowering the water level in the tanks to 20 cm for 30 min giving an effective density of 500 kg/m^3^. Prior to sampling the fish were killed with an overdose of anesthetic (Finquel, Scanvac Årnes, Norway). Skin samples (dorsal side) were collected on the 28^th^ November (time zero; average weight 103g ± 23) and the 2^nd^ (day 1; 99g ± 14), 8^th^ (day 7; 96g ± 19) and 15^th^ (day 14; 96g ± 25) of December. All samples were snap frozen in liquid nitrogen and stored at -80°C.

Ethical statement: This study was approved by the national ethics committee (the Norwegian Food Safety Authority) and carried out in strict accordance with the Norwegian animal welfare act (LOV-2009-06-19-97) and the regulation on the use of animals in research (FOR-2015-06-18-761).

#### Quantitative real-time RT-PCR (qPCR)

Frozen skin sections were transferred to TRIzol (Thermo Fisher Scientific) and homogenized with 2,8 mm zirconium oxide beads (Precellys®24) in a Precellys®24 homogenizer (Precellys®24). RNA was extracted using PureLink™ Pro 96-well purification kit (Thermo Fisher Scientific) with on-column-DNase (Qiagen) digestion. RNA concentration and quality was measured with NanoDrop (Thermo Fisher Scientific). Synthesis of cDNA was performed on 500 ng RNA with SuperScript® VILO cDNA Synthesis Kit and Master Mix (Thermo Fisher Scientific). Primers were designed with Primer3 (v. 0.4.0) [30, 31] (Table 1). QPCR was performed in duplicates in 96-well optical plates on a LightCycler 480 (Roche Diagnostics). Each well had a final reaction volume of 12 μl, using 6 μl SYBR® Green Master Mix (Roche Diagnostics), 5 μl of 1:10 diluted cDNA and primers at a final concentration of 0.42 μM.

**Table 1:** Primers and general information.

**RefSeq gene name**	**Symbol**	**Gene–ID**	**Main tissue of transcription**	**Chromosome**	**Forward primer**	**Reverse primer**	**AL/E**^**a**^
muc5ac-like	*muc5ac*.*1*	XP_013982550.1	Skin	11	gacctgctctgtggaaggag	agcacggtgaattcagttcc	120/ 1.9
muc5ac-like^b^	*muc5ac*.*2*	XP_013981532.1	Gill	10	ttttctcagttgccgctttt	agtcggagcccataagaggt	92/ 1.8
muc5ac-like	*muc5ac*.*3*	XP_014031311.1	Pyloric ceca	26	-	-	
muc5ac-like^b^	*muc5ac*.*4*	XP_014037804.1	Gill	Scaffold	ttttctcagttgccgctttt	agtcggagcccataagaggt	92/ 1.8
muc5b-like	*muc5b*	XP_014031349.1	Skin	26	attaagagcgatgtcttcacagc	aagcacatgagtctctcacacaa	85/ 1.9
muc2-like^c^	*muc2*.*1*	XP_014025861.1	Intestine	23	gagtgggctctcagatccag	gatgatgcggacggtagttt	99/ 1.9
muc2-like^c^	*muc2*.*2*	XP_014040158.1	Intestine	Scaffold	gagtgggctctcagatccag	gatgatgcggacggtagttt	99/ 1.9
**Reference genes**							
Elongation factor 1 alfa	*elf1a*	AF321836			caccaccggccatctgatctacaa	tcagcagcctccttctcgaacttc	78/ 1.9
Eukaryotic translation initiation factor 3	*etif3*	DW542195			caggatgttgttgctggatggg	acccaactgggcaggtcaaga	102/ 1.9

^a^AL/E: cDNA amplicon length/Primer efficiency

^b^ Primers bind *muc5ac*.*2* and *muc5ac*.*4*

^c^ Primers bind *muc2*.*1* and *muc2*.*2*

Quantification cycle (Ct) values were calculated using the second derivative method (stress experiments) or the fit-points method (tissue specific transcription study) in the LightCycler 480 software (version 1.5.0.39). Duplicates showing a Ct difference < 0.5 were removed from further analysis. The efficiency of the qPCR reactions were estimated for all primer pairs by five times 1:2 dilution series. Two reference genes were evaluated for stability using the web-based comprehensive tool RefFinder which integrates the computational programs geNorm, Normfinder, BestKeeper and the comparative delta-Ct method [32]. The most stable reference gene was *etif3* and this gene was used in the normalization procedure.

The specificity of the reactions was verified by analysis of the melting curves, electrophoresis and sequencing of the amplified PCR products. Prior to sequencing, fragments were amplified with AmpliTac Gold® DNA polymerase (Applied Biosystems) and dNTPs (Promega) in a 10μl PCR reaction. ExoSap IT (Affymetrix) was used to remove excess primers and nucleotides according to the manufactures instructions. The sequencing reaction was run using a BigDye Terminator v.1.1 Cycle Kit (Applied Biosystems) and cleaned with a BigDye Xterminator Purification Kit (Applied Biosystems). Sanger sequencing was performed on 3130xl Genetic analyzer (Applied Biosystems) and sequences were analyzed with Sequence Scanner v2.0 (Applied Biosystems).

#### Data analysis

All data analysis were performed in R (www.r-project.org). In the tissue specific transcription study the delta Ct-values were averaged for each gene and tissue. The values were inverted and subsequently plotted as a heat map where the transcription in each tissue is presented relative to each other. Each square represents the average delta-Ct value of three individual animals (n = 2 for esophagus, stomach and ovarium).

Experiment 1: Significant changes in transcription were estimated by fitting a linear model to the delta-Ct values, separately for each gene and tissue. Groups showing a p-value < 0.05 were considered to be significantly different from the control group.

Experiment 2: Significant changes in transcription were estimated by fitting a linear model to the delta-Ct values for each gene. Groups showing a p-value < 0.05 were considered to be significantly different from day 0.

## Results and discussion

We are just beginning to understand the complex nature of mucosal protection, a system which consists of both bioactive compounds and physical protection. The main constituents of the mucus matrix are the mucin proteins and the present study is the first comprehensive characterization of Atlantic salmon mucins, describing domain structure, phylogenetic relationship, tissue-specific transcription and transcriptional regulation in response to stress conditions relevant for aquaculture production of salmonids. Seven unique mucins were identified as secreted gel-forming mucins in the Atlantic salmon reference genome, including two mucins annotated as *muc2* and five mucins annotated as *muc5*. In short, this study contributes to the understanding of mucosal protection which may provide a direct pathway to enhance the health and welfare of farmed fish.

### Identification and structural characterization of putative mucin genes

**Fig 2:**
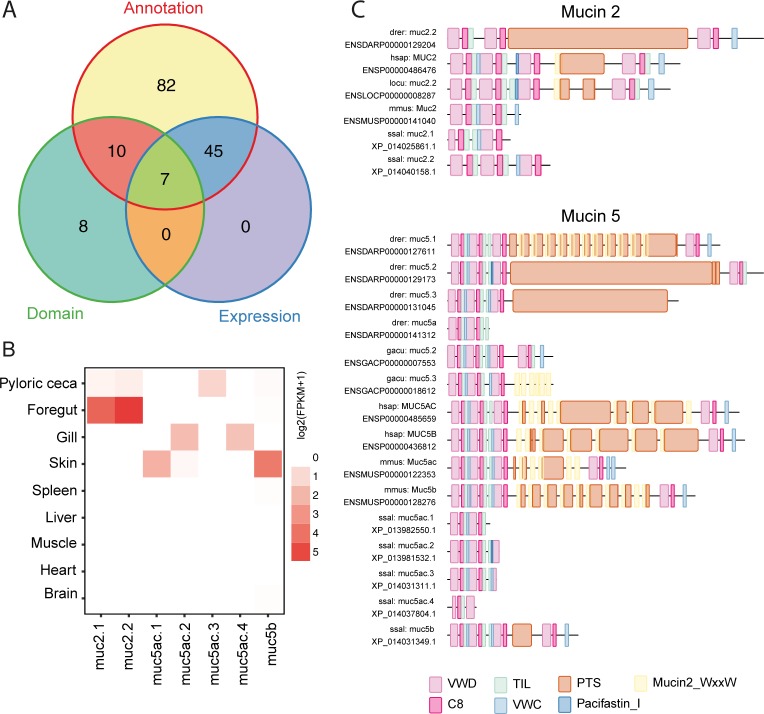
Identification of seven putative mucin genes, with annotation, transcription (public RNAseq data) and protein architecture. **A** According to RefSeq annotation, 144 genes were annotated as mucins, out of these 52 of were considered to be transcribed, whereas 25 genes had a minimum domain structure of VWD-C8-TIL. Seven mucin genes were found using the combination of these three approaches. **B** Protein architecture of the seven putative Atlantic salmon (*Salmo salar*, **ssal**), mucin genes. For comparison, the protein architecture of Muc2 and Muc5 from stickleback (*Gasterosteus aculeatus*, **gacu**), zebrafish (*Danio rerio*, **drer**), mouse (*Mus musculus*, **mmouse**), human (*Homo sapiens*, **hsap**) is also presented in the figure. **C** Transcription of the seven putative mucin genes in the tissues; Pyloric ceca, foregut, skin, pancreas, gill, spleen, liver, muscle heart and brain. The data is based on a public RNA-Seq data set. FPKM values as indicated in the figure.

Our search for Atlantic salmon mucin genes began with the identification of all the genes annotated as mucins in the recently released Atlantic salmon genome [25]. This approach identified 144 potential mucins (Fig 2A). Further, transcription profiles for the 144 genes were analyzed with a publicly available RNA-seq dataset [25]; from this it was evident that 52 genes were transcribed in one or more tissues, while the remaining 92 genes had very low or no detectable transcription (FPKM < = 1). The sequences of all the annotated mucins were then analysed for domain structure. This resulted in a total of 25 genes that contained the VWD-C8-TIL domain structure. Combining these three approaches, annotation, domain structure and transcription, seven mucin genes were identified, two genes in the *muc2* family and five genes in the *muc5* family (Fig 2A). In order to separate between the different mucin genes within the same family, a symbol was assigned to each of the genes (Table 1).

The domain structure of the seven putative mucin genes is presented together with the domain structure of mucins from other species (Fig 2B). Here we see that only Muc5b had the domain architecture of 3X(VWD-C8-TIL-VWC)-PTS-(VWD-C8-TIL), which is expected for the Muc5 family [17]. The remaining mucins lacked the PTS domain and one or more of the VWD domains. The PTS-domain is where the repetitive sequences are found, which often cause problems for sequencing and alignments [21]. In addition, PTS domains are only loosely defined and here we used the definition of a PTS domain as described in Lang et. al. 2004, meaning that there may be areas suitable for glycosylation in the sequence. Lastly, we examined the transcription pattern from the seven putative mucin genes (Fig 2C). As expected, the mucin genes were mainly transcribed in the mucosal tissues. The genes in the *muc2* family had a similar tissue distribution, being mainly transcribed in the foregut and pyloric caeca while the genes in the *muc5* family had a wider tissue distribution. *Muc5ac*.*1* and *muc5b* were mainly transcribed in the skin, *muc5ac*.*3* in pyloric caeca and *muc5ac*.*2* and *muc5ac*.*4* in the gill. These results match the profile of secreted gel-forming mucins in other species (discussed in the qPCR section below). In conclusion, seven putative mucin genes were identified using annotation, transcription and domain structure approaches. Both the transcription profile and the domain structure of these genes matches the profile of mucin genes in other species which supports our hypothesis that these genes are genuine mucins.

**Fig 3:**
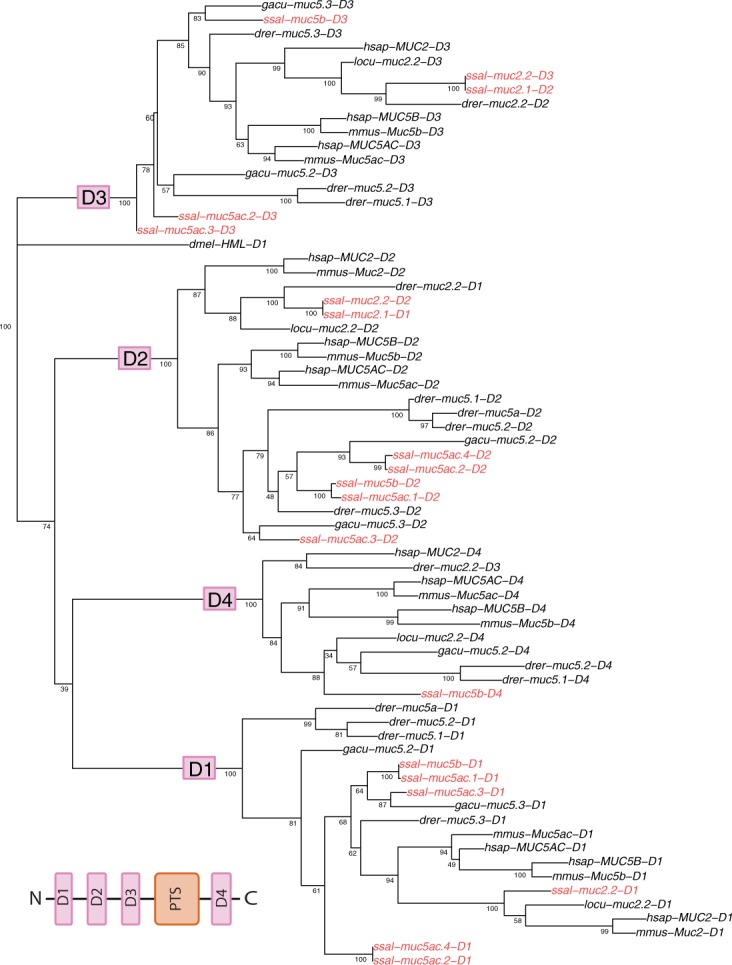
Phylogenetic tree of the identified Atlantic salmon mucins. The main branches represents clustering of the VWD domains, referred to as D1 to D4 based on their N-terminal position, as indicated in the insert figure. The Atlantic salmon (*Salmo salar*, **ssal**) VWD domains are highlighted in red, in total 19. In addition, VWD domains from several species are included in the tree, including stickleback (*Gasterosteus aculeatus*, **gacu**), spotted gar (*Lepisosteus* gacu, **locu**) zebrafish (*Danio rerio*, **drer**), mouse (*Mus musculus*, **mmouse**), human (*Homo sapiens*, **hsap**). The tree is rooted to the VWD domain found in Hemolectin of fruit fly (*Drosophila melanogaster*, **dmel**), a non-vertebrate mucin orthologue [21].

The major purpose of constructing the phylogenetic trees was to verify the classification of the predicted mucins. The phylogenetic tree was built based on the alignments of the VWD domains (Fig 3), as these domains represent the conserved areas in the genetic sequence [17]. In total, 19 Atlantic salmon VWD domains were aligned, corresponding to the number of the identified VWD domains found in the seven putative mucin sequences (Fig 2C). The Atlantic salmon domains were aligned with VWD domains both from fish species, and more distant related species such as mice and human. The Atlantic salmon mucins reflected the evolutionary relationships among species. Further, the tree clustered VWD domains from the Muc2 family together and the Muc5 family together, demonstrating a close interspecies relationship between these domains. Thus, we are quite certain that the seven putative mucin genes were annotated as the correct mucin variant.

Lastly we looked at the relationship between VWD domains with similar N-terminal positions. The Atlantic salmon VWD domains from the Muc5 family and Muc2.2 clustered together with VWD domains with similar N-terminal positions. This suggests that VWD domains with similar N-terminal positions are related to each other, and that the order of the domains are conserved in the analysed species. Muc2.1 was an exception to this, as the D1 domain was more closely related to the D2 family, and the D2 domain was more closely related to the D3 family. An explanation for this may be because one N-terminal VWD domain was missing in the sequence (Fig 2C). Overall, this close relationship between VWD domains with similar N-terminal position suggests that the seven predicted Atlantic salmon mucin sequences were assembled in a correct way by the RefSeq genome assembly.

### Mucin transcription in mucosal tissues

Based on the seven putative mucin gene sequences, qPCR primers were designed to examine their transcription in a wide range of tissues from post-smolt salmon reared in seawater. Due to their highly repetitive nature and sequence similarity, it was difficult to obtain specific primers for all the seven mucin genes. Finally, four primer pairs were used in this study, two of these pairs bound to specific mucin sequences (*muc5ac*.*1* and m*uc5b*) and two pairs did not. One primer pair bound both copies of *muc2* (*muc2*.*1* and *muc2*.*2*) and the other primer pair bound to both *muc5ac*.*2* and *muc5ac*.*4*.

**Fig 4:**
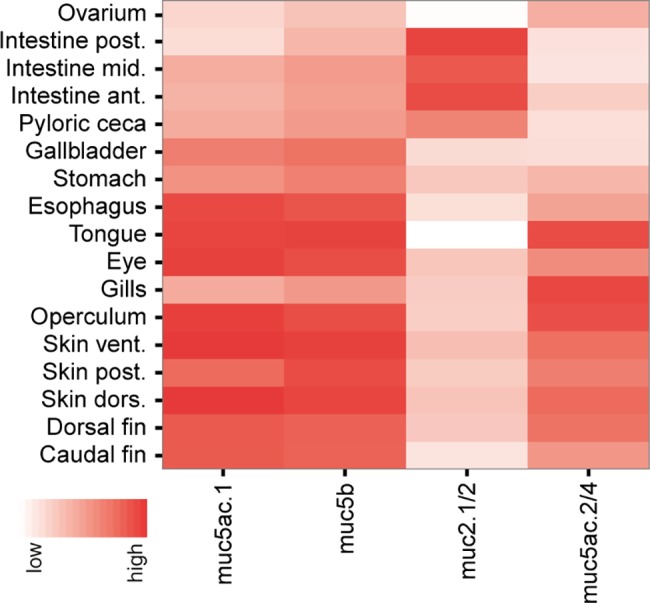
Mucin transcription levels in mucosal tissues, measured with qPCR. Transcription of *muc2*.*1/2*, *muc5ac*.*1*, *muc5ac*.*2/4 and* m*uc5b* in a set of different tissues. Each square represent the inverted mean of the delta-Ct-values n = 3 (n = 2 esophagus, stomach, ovarium).

QPCR analysis of different tissue sections from the skin, fins and gastrointestinal tract showed that *muc2*.*1/2* was most strongly transcribed in the intestine with similar transcription levels between the anterior, middle and posterior parts (Fig 4). Transcription of *muc2*.*1/2* was also moderate in the pyloric caeca, low in the other analyzed organs and absent in the ovarium, tongue and caudal fin. Tissue transcription of the *muc5* family varied with the different genes. *Muc5ac*.*1* and *muc5b* had similar transcription patterns, with high transcription levels in the skin, fins, eye, esophagus and tongue. This transcription pattern was different from *muc5ac*.*2/4* that were highly transcribed in the gill and ovarium, and to a lower level in the skin. These results were concordant with the transcription levels that were obtained from the RNA-seq data, suggesting that the different genes in the *muc5* family have tissue-specific transcription patterns. Similar tissue distribution and transcription patterns were also observed in other species. For example in zebrafish, different *muc5ac* genes were transcribed in skin, gill, pharynx and esophagus [33]. In common carp, *muc5b* transcripts were detected in the skin [34] and in humans different Muc5ac transcripts were transcribed in the respiratory tract, stomach, cervix and eye [35]. The transcription profile of the *muc2* family were also similar to the transcription profiles found in other species. *Muc2* seems to be dominantly transcribed in the gastrointestinal tract of gilthead sea bream (*Sparus aurata*) [36], zebrafish [33], common carp (*Cyprinus carpio*) [34] as well as in humans [37].

In a practical manner, tissue specific distribution of the mucin genes may be important in order to understand host pathogen interactions in mucosal tissues. Previous studies have shown that bacteria, such as *Aeromonas salmonicida* binds mucins isolated from the intestinal tract to a greater extent than skin mucins [38]. Further, it is also shown that the glycosylation pattern of mucins is different in the skin and intestine of Atlantic salmon [39]. Thus, knowledge on the tissue distribution of different mucins could explain the observed differences between resistance and susceptibility for infectious diseases. In conclusion, genes in the Atlantic salmon *muc5* family have a wide tissue distribution, while genes in *muc2* family were mainly transcribed in the intestinal regions. These results are in concordance with the transcription profiles of *muc2* and *muc5* in other teleost and mammalian species. Our results bring us one step closer in understanding of the biological effect of the different mucin families.

### Mucin transcription in gill, intestine and skin after acute handling stress

**Fig 5:**
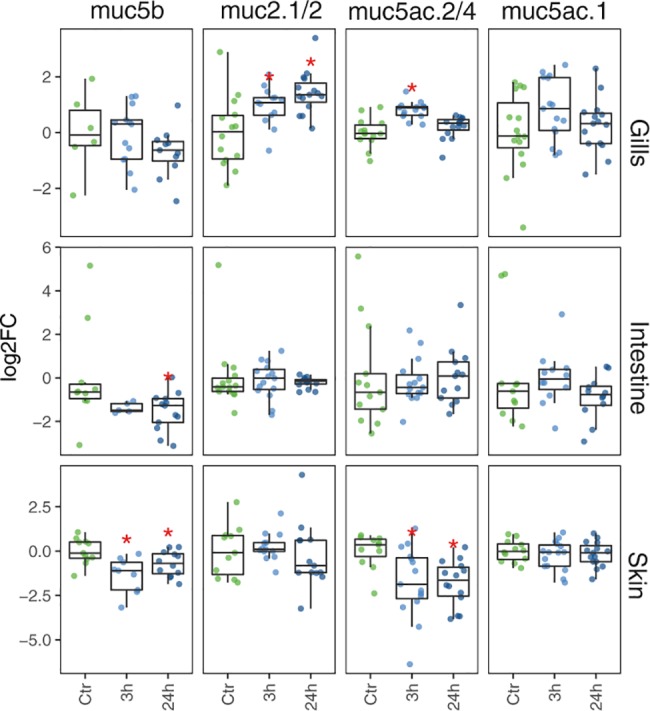
Changes in mucin transcription in response to an acute stressor. Differential transcription of mucin genes in gill, middle intestine and skin in response to handling stress (n = 15). On the X-axis, control (C), three hours post stress (h3) and twenty-four hours post stress (h24). The box-plot representation shows the median value of mRNA transcription (bold line), the lower and upper limits of each box representing the first and third quartiles, respectively. Whiskers represent the limits of extreme measurements. Transcription is displayed as log_2_ fold changes relative to the mean transcription of the mucin gene in the control group of the respective tissue. A red asterisk indicates that the marked group is significantly different compared to the control group (t.test; p.value < 0.05).

Short-term (24 h) effects on mucin transcription in large post-smolts (~300 g) after acute handling stress was examined in the main mucosal tissues; gill, mid-intestine and skin. Results showed that the stressor led to different transcription patterns for the respective mucins depending on tissue and gene (Fig 5). In the gill, stress significantly induced transcription of *muc2*.*1*/*2* (0.94 to 1.43 fold) after 3 and 24 h. Hence, while being transcribed mainly in the intestinal region (Fig 4), *muc2*.*1/2* seems to have a role during stress-induced responses in the gill. Transcription of *muc5ac*.*2/4* also increased significantly 0.8 fold, 3 h post stress, while returning to base-line levels after 24 h (Fig 5). These genes had the highest transcription levels in the gill (Figs 2B and 4), and thus seems to be important for regulating mucus production both under normal (steady-state) and perturbed conditions. In the mid-intestine, handling stress had generally little impact on gene transcription, and only *muc5b* had altered transcription levels, being significantly down-regulated -1.54 fold after 24 h. A similar response was observed in the skin, with *muc5b* being significantly -1.37 and -0.71 fold down-regulated 3 and 24 h post stress and *muc5ac*.*2/4* being significantly -1.74 and -1.8 fold down-regulated at 3h and 24h compared to pre-stress levels.

The reason why the mucin transcription is differentially regulated in different tissues may be due to the metabolic costs of stress [40]. Gills are the primary organ for respiration, and handling stress dramatically increases oxygen consumption [41, 42]. Thus, we suggest that increased mucin transcription in the gill may be an associated effect of increased respiration. On the other hand, the observed decreased transcription in the skin and intestine could be an energy saving response. Thus, the observed differentially transcription patterns may demonstrate a differentially coping mechanism balancing the energy demand in the different tissues.

Further, we see that mucin genes belonging to the same gene family is differentially regulated in the same tissue. *Muc5b* and muc5ac.2/4 are down regulated by handling stress in the skin, whereas *muc5ac*.*1* is unaffected. Apart from *muc5ac*.*1*, of which the transcription was unaffected upon stress in all three tissues, the respective mucins could represent tissue-specific biomarkers for evaluation of effects of handling stress such as transportation and vaccination.

The practical aspects of these observations should also be taken into account, as a non-intact mucus layer is associated with increased risk of secondary infections [43, 44]. Recently high mortalities are reported as a result of mechanical de-lousing, which both stresses the fish and cause damage to the skin and the mucus layer [45]. Here we show that it takes more than 24h before the mucin transcription returns to basal levels in the skin and intestine. If the fish is exposed to rough handling procedures, such as de-lousing, the fish should be given appropriate time to recover (> 24h) in order to restore the protective mucus layers.

### Mucin transcription in the skin after intensive rearing and confinement stress

**Fig 6:**
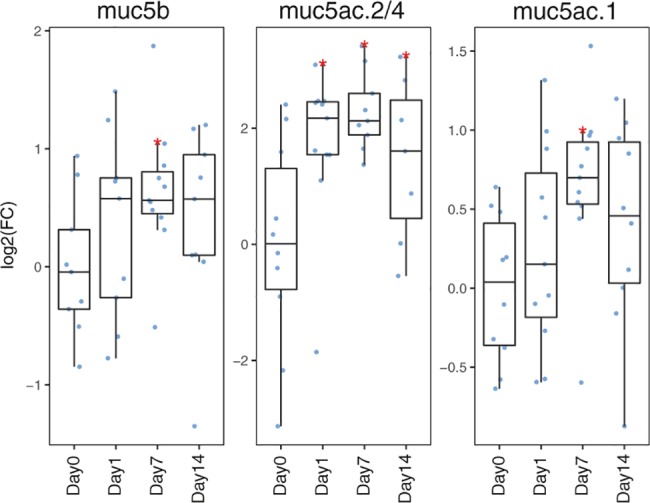
Mucin transcription in the skin in response to intensive rearing and confinement stress. Differential transcription of mucin genes in the skin in response to confinement stress (n = 12). Day 0 represent samples taken three days prior to introduction of confinement stress and day 1, 7 and 14 represent time after introduction to confinement stress. Statistics as in Fig 5.

Mucin transcription in the skin of post-smolts reared under intensive conditions (i.e. high biomass) followed by confinement stress was examined over a period of 14 days.Transcription of *muc5ac*.*2/4* increased significantly already after 24 h and kept up-regulated until the end of the experiment (Fig 6). Similar induced transcription was observed for *muc5b* and *muc5ac*.*1*, but at lower levels and a slower pace, being significantly upregulated first after 7 days and slightly declining thereafter (Fig 6). These findings are in line with previous results showing increased transcription of mucin-like genes in the skin with increasing fish density [46].

Thus, the two experiment that was conducted had different impact on mucin transcription in the skin. Netting and air exposure led to a reduction in mucin transcription, whereas confinement stress followed by high rearing intensities led to increased mucin transcription, indicating that transcription may be differentially regulated by different stressors. In the first experiment, instead of investing energy into the mucus production, which is energy demanding given the large size and heavy glycosylation of these proteins, energy could be allocated to metabolic processes such as glycogenesis which is required for this high energy demanding period. In the second experiment, the fish is exposed to a chronic stressor. In most cases, when fish are exposed to a stressor for a long period they will adapt to the condition [47]. The increased transcription of mucin genes could therefore be an adaptive response to long term stressful conditions. It is also likely that increased fish densities would lead to increased contact between fish and potential abrasion that could stimulate mucin production. High fish densities will also change the water quality parameters in the tank, which also could be a reasonable explanation for the increased mucin transcription in the skin. It is therefore unknown whether the observed increase in mucin transcription in the second experiment was due to more persistent changes in water quality parameters in the tank, skin abrasion or as a specific response to the confinement stress itself.

Future studies should focus on separating the effects of relevant environmental and biological factors on mucin production and transcriptional regulation, in order to further evaluate the application of these mucins as biomarkers for aquaculture-relevant perturbations.

## Conclusion

Seven putative mucin genes were identified in the Atlantic salmon genome. Their transcription patterns show that the tissue-specific transcription pattern of Atlantic salmon *muc2* and *muc5* families are similar to those in other species. Furthermore, the results from two controlled stress experiments show that mucin transcription is regulated in response to aquaculture-relevant stressors, but the response varies depending on the type of stressor. Acute handling stress by netting led to increased mucin transcription in the gill, while it decreased in the skin and intestine. Conversely, long-term intensive rearing conditions and confinement stress led to increased mucin transcription in the skin. Our results will provide an important foundation for better understanding of mucin biology and mucosal health, and their usefulness as biomarkers for aquaculture applications.

## Supporting information

S1 File Supplementary data containing qPCR-raw data and additional primer information, as well as information about the sequences used for building the phylogenetic tree. (XLSX)

## References

[1] Shephard K. Functions for fish mucus. Rev Fish Biol Fisheries. 1994;4(4):401–29. 10.1007/BF00042888

[2] McGuckin MA, Lindén SK, Sutton P, Florin TH. Mucin dynamics and enteric pathogens. Nature Reviews Microbiology. 2011;9(4):265–78. 10.1038/nrmicro2538 21407243

[3] Kufe DW. Mucins in cancer: function, prognosis and therapy. Nature Reviews Cancer. 2009;9(12):874–85. 10.1038/nrc2761 19935676 PMC2951677

[4] Rose MC, Voynow JA. Respiratory tract mucin genes and mucin glycoproteins in health and disease. Physiol Rev. 2006;86(1):245–78. Epub 2005/12/24. 10.1152/physrev.00010.2005 . 16371599

[5] Barton BA, Iwama GK. Physiological changes in fish from stress in aquaculture with emphasis on the response and effects of corticosteroids. Annual Review of Fish Diseases. 1991;1:3–26.

[6] Segner H, Sundh H, Buchmann K, Douxfils J, Sundell KS, Mathieu C, et al Health of farmed fish: its relation to fish welfare and its utility as welfare indicator. Fish Physiology and Biochemistry. 2012;38(1):85–105. 10.1007/s10695-011-9517-9 21681416

[7] Sundh H, Kvamme BO, Fridell F, Olsen RE, Ellis T, Taranger GL, et al Intestinal barrier function of Atlantic salmon (*Salmo salar L*.) post smolts is reduced by common sea cage environments and suggested as a possible physiological welfare indicator. BMC physiology. 2010;10(1):1. 21062437 10.1186/1472-6793-10-22 PMC2992494

[8] Esteban M. An Overview of the Immunological Defenses in Fish Skin. ISRN Immunology. 2012;2012:29 10.5402/2012/853470

[9] Pittman K, Sourd P, Ravnøy B, Espeland Ø, Fiksdal IU, Oen T, et al Novel method for quantifying salmonid mucous cells. Journal of Fish Diseases. 2011;34(12):931–6. 10.1111/j.1365-2761.2011.01308.x 22004586

[10] Zuchelkowski EM, Lantz RC, Hinton DE. Effects of acid-stress on epidermal mucous cells of the brown bullhead *Ictalurus nebulosus* (LeSeur): A morphometric study. The Anatomical record. 1981;200(1):33–9. 10.1002/ar.1092000104 7258692

[11] Zuchelkowski EM, Pinkstaff CA, Hinton DE. Mucosubstance histochemistry in control and acid-stressed epidermis of brown bullhead catfish, *lctalurus nebulosus* (LeSueur). The Anatomical record. 1985;212(4):327–35. 10.1002/ar.1092120402 4073548

[12] Vatsos IN, Kotzamanis Y, Henry M, Angelidis P, Alexis M. Monitoring stress in fish by applying image analysis to their skin mucous cells. European journal of histochemistry: EJH. 2010;54(2):e22 Epub 2010/06/19. 10.4081/ejh.2010.e22 ; PubMed Central PMCID: PMCPmc3167306. 20558343 PMC3167306

[13] Ledy K, Giamberini L, Pihan JC. Mucous cell responses in gill and skin of brown trout (*Salmo trutta*) fario in acidic, aluminium-containing stream water. Dis Aquat Organ. 2003;56(3):235–40. Epub 2003/12/12. 10.3354/dao056235 . 14667035

[14] Van Der Marel M, Caspari N, Neuhaus H, Meyer W, Enss ML, Steinhagen D. Changes in skin mucus of common carp, *Cyprinus carpio L*., after exposure to water with a high bacterial load. Journal of Fish Diseases. 2010;33(5):431–9. 10.1111/j.1365-2761.2010.01140.x 20298445

[15] Bols NC, Brubacher JL, Ganassin RC, Lee LEJ. Ecotoxicology and innate immunity in fish. Developmental & Comparative Immunology. 2001;25(8–9):853–73. 10.1016/S0145-305X(01)00040-4. 11602200

[16] Hoole D, Bucke D, Burgess P, Wellby I, (Eds). Diseases of Carp and Other Cyprinid Fishes Hoole D, Bucke D, Burgess P, Wellby I, editors: Wiley-Blackwell; 2001 280 p.

[17] Lang T, Klasson S, Larsson E, Johansson ME, Hansson GC, Samuelsson T. Searching the evolutionary origin of epithelial mucus protein components–mucins and FCGBP. Molecular biology and evolution. 2016:msw066. 10.1093/molbev/msw066 PMC4948705 27189557

[18] Perez-Vilar J, Hill RL. The structure and assembly of secreted mucins. Journal of Biological Chemistry. 1999;274(45):31751–4. 10542193 10.1074/jbc.274.45.31751

[19] Allen A, Hutton DA, Pearson JP, Sellers LA. Mucus glycoprotein structure, gel formation and gastrointestinal mucus function. Ciba Foundation symposium. 1984;109:137–56. Epub 1984/01/01. . 6394242 10.1002/9780470720905.ch10

[20] Strous GJ, Dekker J. Mucin-Type Glycoproteins. Critical Reviews in Biochemistry and Molecular Biology. 1992;27(1–2):57–92. 10.3109/10409239209082559 . 1727693

[21] Lang T, Hansson GC, Samuelsson T. Gel-forming mucins appeared early in metazoan evolution. Proceedings of the National Academy of Sciences. 2007;104(41):16209–14. 10.1073/pnas.0705984104 PMC2042186 17911254

[22] Micallef G, Bickerdike R, Reiff C, Fernandes JO, Bowman A, Martin SM. Exploring the Transcriptome of Atlantic Salmon (Salmo salar) Skin, a Major Defense Organ. Marine Biotechnology. 2012;14(5):559–69. 10.1007/s10126-012-9447-2 22527268

[23] Eddy SR. Accelerated Profile HMM Searches. PLoS Comput Biol. 2011;7(10):e1002195 10.1371/journal.pcbi.1002195 22039361 PMC3197634

[24] Lang T, Alexandersson M, Hansson GC, Samuelsson T. Bioinformatic identification of polymerizing and transmembrane mucins in the puffer fish Fugu rubripes. Glycobiology. 2004;14(6):521–7. 10.1093/glycob/cwh066 15044386

[25] Lien S, Koop BF, Sandve SR, Miller JR, Kent MP, Nome T, et al The Atlantic salmon genome provides insights into rediploidization. Nature. 2016;533(7602):200–5. 10.1038/nature17164 27088604 PMC8127823

[26] Aken BL, Ayling S, Barrell D, Clarke L, Curwen V, Fairley S, et al The Ensembl gene annotation system. Database. 2016;2016:baw093. 10.1093/database/baw093 PMC4919035 27337980

[27] Katoh K, Standley DM. MAFFT Multiple Sequence Alignment Software Version 7: Improvements in Performance and Usability. Molecular Biology and Evolution. 2013;30(4):772–80. 10.1093/molbev/mst010 PMC3603318. 23329690 PMC3603318

[28] Schliep KP. phangorn: phylogenetic analysis in R. Bioinformatics (Oxford, England). 2011;27(4):592–3. 10.1093/bioinformatics/btq706 PMC3035803. 21169378 PMC3035803

[29] Whelan S, Goldman N. A general empirical model of protein evolution derived from multiple protein families using a maximum-likelihood approach. Mol Biol Evol. 2001;18(5):691–9. Epub 2001/04/25. . 11319253 10.1093/oxfordjournals.molbev.a003851

[30] Untergasser A, Cutcutache I, Koressaar T, Ye J, Faircloth BC, Remm M, et al Primer3—new capabilities and interfaces. Nucleic acids research. 2012;40(15):e115–e. 10.1093/nar/gks596 22730293 PMC3424584

[31] Koressaar T, Remm M. Enhancements and modifications of primer design program Primer3. Bioinformatics (Oxford, England). 2007;23(10):1289–91. Epub 2007/03/24. 10.1093/bioinformatics/btm091 . 17379693

[32] Xie F, Xiao P, Chen D, Xu L, Zhang B. miRDeepFinder: a miRNA analysis tool for deep sequencing of plant small RNAs. Plant molecular biology. 2012 Epub 2012/02/01. 10.1007/s11103-012-9885-2 . 22290409

[33] Jevtov I, Samuelsson T, Yao G, Amsterdam A, Ribbeck K. Zebra fish as a model to study live mucus physiology. Sci Rep. 2014;4. 10.1038/srep06653 PMC4200417 25323747

[34] van der Marel M, Adamek M, Gonzalez SF, Frost P, Rombout JHWM, Wiegertjes GF, et al Molecular cloning and expression of two β-defensin and two mucin genes in common carp (*Cyprinus carpio L*.) and their up-regulation after β-glucan feeding. Fish & shellfish immunology. 2012;32(3):494–501. 10.1016/j.fsi.2011.12.008. 22227003

[35] Linden SK, Sutton P, Karlsson NG, Korolik V, McGuckin MA. Mucins in the mucosal barrier to infection. Mucosal Immunol. 2008;1(3):183–97. 10.1038/mi.2008.5 19079178 PMC7100821

[36] Perez-Sanchez J, Estensoro I, Redondo MJ, Calduch-Giner JA, Kaushik S, Sitja-Bobadilla A. Mucins as diagnostic and prognostic biomarkers in a fish-parasite model: transcriptional and functional analysis. PloS one. 2013;8(6):e65457 Epub 2013/06/19. 10.1371/journal.pone.0065457 ; PubMed Central PMCID: PMCPmc3680472. 23776483 PMC3680472

[37] Kim Y, Ho S. Intestinal Goblet Cells and Mucins in Health and Disease: Recent Insights and Progress. Curr Gastroenterol Rep. 2010;12(5):319–30. 10.1007/s11894-010-0131-2 20703838 PMC2933006

[38] Padra JT, Sundh H, Jin C, Karlsson NG, Sundell K, Lindén SK. Aeromonas salmonicida Binds Differentially to Mucins Isolated from Skin and Intestinal Regions of Atlantic Salmon in an N-Acetylneuraminic Acid-Dependent Manner. Infection and immunity. 2014;82(12):5235–45. 10.1128/IAI.01931-14 25287918 PMC4249282

[39] Jin C, Padra JnTs, Sundell K, Sundh H, Karlsson NG, Lindeén SK. Atlantic salmon carries a range of novel O-glycan structures differentially localized on skin and intestinal mucins. Journal of proteome research. 2015;14(8):3239–51. 10.1021/acs.jproteome.5b00232 26066491

[40] Iwama GK. Stress in fish. Annals of the New York Academy of Sciences. 1998;851(1):304–10.

[41] Radull J, Kaiser H, Hecht T. Stress-related changes in the metabolic rate of juvenile spotted grunter, Pomadasys commersonnii (*Haemulidae*, *Pisces*). Marine and freshwater research. 2002;53(2):465–9.

[42] Barton BA, Schreck CB. Metabolic Cost of Acute Physical Stress in Juvenile Steelhead. Transactions of the American Fisheries Society. 1987;116(2):257–63. 10.1577/1548-8659(1987)116<257:MCOAPS>2.0.CO;2

[43] Svendsen YS, Bøgwald J. Influence of artificial wound and non-intact mucus layer on mortality of Atlantic salmon (*Salmo salar L*.) following a bath challenge with *Vibrio anguillarum* and *Aeromonas salmonicida*. Fish & shellfish immunology. 1997;7(5):317–25.

[44] Madetoja J, Nyman P, Wiklund T. Flavobacterium psychrophilum, invasion into and shedding by rainbow trout *Oncorhynchus mykiss*. Dis Aquat Organ. 2000;43(1):27–38. Epub 2000/12/29. 10.3354/dao043027 . 11129378

[45] Gismervik K, Nielsen KV, Lind M, Viljugrein H. Mekanisk avlusing med FLS-avlusersystem dokumentasjon av fiskevelferd og effekt mot lus. Veterinærinstituttets rapportserie 6–2017: 2017.

[46] Sveen LR, Timmerhaus G, Torgersen JS, Ytteborg E, Jørgensen SM, Handeland S, et al Impact of fish density and specific water flow on skin properties in Atlantic salmon (*Salmo salar L*.) post-smolts. Aquaculture. 2016;464:629–37. 10.1016/j.aquaculture.2016.08.012.

[47] Barton BA. Stress in Fishes: A Diversity of Responses with Particular Reference to Changes in Circulating Corticosteroids1. Integrative and Comparative Biology. 2002;42(3):517–25. 10.1093/icb/42.3.517 21708747

